# Methods of measuring the iridocorneal angle in tomographic images of the anterior segment of the eye

**DOI:** 10.1186/1475-925X-12-40

**Published:** 2013-05-10

**Authors:** Robert Koprowski, Zygmunt Wróbel, Sławomir Wilczyński, Anna Nowińska, Edward Wylęgała

**Affiliations:** 1 Department of Biomedical Computer Systems, Institute of Computer Science, University of Silesia, Będzińska 39 Str, Sosnowiec, 41-200, Poland; 2 Department of Biophysics, School of Pharmacy, Medical University of Silesia, Jednosci 8 Str, Sosnowiec, 41-200, Poland; 3 Oddział Okulistyki Okręgowego Szpitala Kolejowego w Katowicach, Okręgowy Szpital Kolejowy w, Katowicach, Poland

**Keywords:** Eye, Image processing, Iridocorneal angle, OCT

## Abstract

**Introduction:**

This paper presents the problem of automatic measurement of the iridocorneal angle in tomographic images of the anterior segment of the eye. It includes the results of the comparison of well-known methods for measuring the iridocorneal angle with new methods, proposed in this paper. All these methods concern tomographic image analysis and processing.

**Material and method:**

In total, approximately 100’000 tomographic images (from about 6’000 patients) were analysed. They were obtained using two devices: SOCT Copernicus (Optopol Tech. SA, Zawiercie, Poland) and Visante OCT (Carl Zeiss Meditec, Inc, Dublin, California, USA). The patients, aged 12 to 78 years with varying degrees of the iridocorneal angle pathology, were from the region of Silesia, Poland. The images were in DICOM or RAW formats and analysed in the software developed by the authors for the purposes of this study.

**Results:**

The results indicate that the measurement method proposed by the authors, which is based on the calculation of the minimum distance between the iris and the cornea in the adopted area, is the most accurate. For this method sensitivity was 0.88, specificity 0.89 and the area under the Receiver Operating Characteristic curve (*AUC*) was 0.88. The other known methods for measuring the iridocorneal angle gave worse results, that is, for example, for the measurement of the distance between the iris and the cornea *AUC* = 0.87, sensitivity = 0.86 and specificity = 0.71. For another well-known method of measuring the iridocorneal angle *AUC* = 0.77, sensitivity = 0.82 and specificity = 0.61.

**Conclusions:**

The study proved that the proposed method of measuring the minimum distance between the iris and the cornea within the adopted area is the most effective in the classification of the iridocorneal angle in patients with a high degree of pathology of all the compared measurement methods based on tomographic images. However, it requires fully automated measurement.

## Introduction

This paper compares well-known methods for the iridocorneal angle analysis in tomographic images of the anterior segment of the eye with the new ones, suggested by the authors. The iridocorneal angle is the structure responsible for the outflow of aqueous humor from the anterior chamber of the eye. Normal intraocular pressure is determined by the production of aqueous humor by the ciliary epithelium and the rate of humor outflow via two pathways – the trabecular meshwork and the uveoscleral pathway [1,2]. Anatomical anomalies such as the angle narrowing or closure result in impeded outflow and increased intraocular pressure.

**Figure 1:**
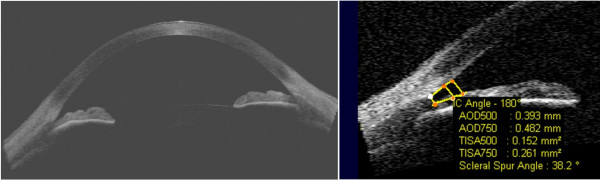
**Tomographic image of the anterior segment of the eye and examples of commercial software operation provided with the Visante OCT device.** In the illustrated case, an operator indicates one point for the scleral spur position (highlighted with a white circle) and the machine adjusts its position and draws the location of consecutive points marked with red circles. The results for the analysed case are *AOD* = 393 μm, *TISA* = 0.152 mm^2^, *TIA* = 38.2°.

**Figure 2:**
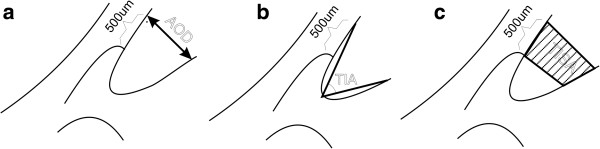
**Methods of measuring the iridocorneal angle. a**) *AOD 500* (Angel Opening Distance) involves measuring a distance between a point of the cornea which is 500 μm away from the scleral spur and the opposite point of the iris. **b**) *TIA* (Trabecular-Iris Angle) involves a direct measurement of the angle. **c**) *TISA 500* (Trabecular-Iris Space Area) involves measuring an area covering 500 μm located in the area bounded by the cornea and the iris.

The iridocorneal angle is situated on the circumference of the anterior chamber between the sides of the cornea and sclera and the base of the iris and the anterior surface of the ciliary body. Currently, a primary diagnostic tool and technique that enables the analysis of the angle structures is gonioscopy which uses contact gonio lenses [3]. The angle is also studied with OCT devices and the technique is called automatic gonioscopy. The main advantage of this method is its non-invasiveness, whereas the advantage of classical gonioscopy is the ability to visualize pathological structures, such as neovascularization or hyperpigmentation of the weave of the trabecular meshwork [4]. The assessment of the iridocorneal angle using OCT Visante involves morphological assessment and a morphometric analysis of the angle parameters. Morphometric measurements can be carried out using the measuring tool "caliper" included in Visante OCT commercial software. The tool is intended to assist manual operation – Figure 1. An operator typically indicates a point on the scleral spur in a OCT image (marked with a white circle in Figure 1) whereas a device adjusts the position of the other points (marked with red circles – in Figure 1). In order to perform these morphometric measurements, it is necessary to manually locate the scleral spur, which is a landmark for determining morphometric measurements, in the scan "ASS". In practice, it often happens that the scleral spur is not visible. According to the results given in paper [5], such a situation occurs in approximately 20% of cases. The known methods *TIA, AOD 500, AOD 750, TISA 500, TISA 750* can be defined on the basis of these morphometric measurements – Figure 2[6] and Table 1.

**Table 1:** Known methods for measuring the iridocorneal angle and their definitions

**Method symbol**	**Method name**	**Definition**
*AOD*	Angle Opening Distance	(Figure 2a) involves measuring a distance between a point of the cornea which is 500 μm away from the scleral spur and the opposite point of the iris
*TIA*	Trabecular-Iris Angle	(Figure 2b) involves a direct measurement of the angle
*TISA*	Trabecular-Iris Space Area	(Figure 2c) involves measuring an area covering 500 μm located in the area bounded by the cornea and the iris

**Figure 3:**
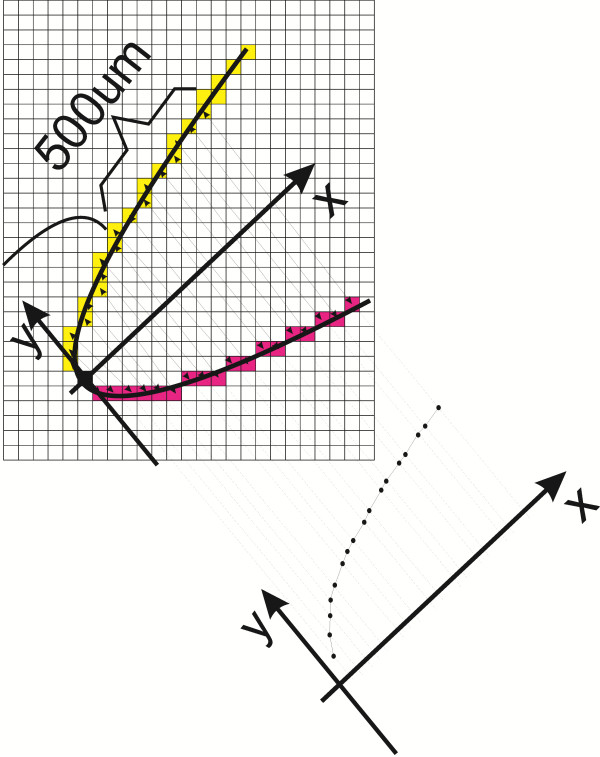
**Measuring principle of the iridocorneal angle with *AOS *(Angel Opening Sequence) suggested by the authors in 2011.** Each iris contour point (in red) is connected to a suitable corneal contour point (in yellow). A division into the appropriate contour is performed based on the point in the largest curvature (shown in black). In this way, a sequence of measurements at various distances from the apex of the measured angle is obtained.

**Figure 4:**
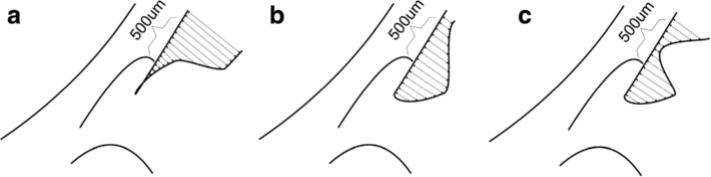
**Various degrees of the iridocorneal angle pathology with marked measurement points for *AOS*.** The presented cases **a**), **b**) and **c**) are not correctly measured with *TIA, TISA* and *AOD*. This is due to both a visible pathology (a variable iris shape) as well as the definition of TIA*, TISA* and *AOD*.

These methods are described in detail in papers [1-3,7]. The values "500" and "750" refer to the distance, expressed in microns, from the scleral spur. As mentioned above, on the basis of the scleral spur location point indicated by an operator, the other characteristic points are drawn automatically. They are necessary to perform calculations for the presented methods, namely *TIA, TISA* and *AOD*. Undoubtedly, this semi-automatic method facilitates operator's work but still it is not done fully automatically. Fully automatic measurement was suggested by the authors in 2011 [7]. It involves the use of information about iridocorneal contours. Each iris contour point is combined with a suitable corneal contour point. A division of contours is performed based on the point of the greatest curvature (point marked in black, the starting point of the coordinate system – Figure 3). In this way, a sequence of measurements at various distances from the apex of the measured angle is obtained – the chart shown in Figure 3. This method, referred to as *AOS* (Angle Opening Sequence), ensures obtaining much more information on the iridocorneal angle when compared to *AOD, TIA* and *TISA*. The varying degree of the iridocorneal angle pathology visible in Figure 4 (especially narrow or closed iridocorneal angle), which is difficult to measure with conventional methods such as *TIA, TISA* and *AOD,* is successfully and reliably evaluated using *AOS*[8]. It is apparent from the examples shown in Figure 4 that a difficulty in a reliable assessment of the iridocorneal angle with the *AOD, TIA* and *TISA* methods lies primarily in a large extent of pathology – distorted sides of the angle, especially of the iris angle. Therefore, in pathological conditions, measurement results of *TISA* and *AOD* are strictly dependent on measurement locations. Slightly less sensitive to this type of pathology is the *TISA* method. However, its practical application is limited due to the area unit (μm^2^) which is less intuitive and difficult to quickly compare with the other methods, *TIA* and *AOD*. In practice, the described methods for measuring the iridocorneal angle, namely *TIA, TISA* and *AOD,* have a number of inconsistencies and irregularities in the interpretation of results. As a consequence, results are not repeatable, reliable and difficult to verify and compare with the model and other doctors’ results. The situation becomes critical when the progress of treatment or the disease progression of a patient diagnosed by different doctors in different medical centres equipped with different types of OCT devices needs to be assessed.

Archiving results is an important issue in practical measurements. In the case of *TIA, TISA* and *AOD*, each measurement is connected with archiving one scalar value, e.g. the angle value measured with *TIA* with a typical accuracy of one decimal place. In the case of *AOS,* a data vector of successive measurements of iridocorneal contour distances is archived. In paper [7] in 2011, the authors presented a new way of recording the results obtained with the *AOS* method, which included the alphabet shown in Table 2.

**Table 2:** Alphabet for the iridocorneal angle description proposed by the authors in 2011

**Symbols**	**Function**
**/**	increasing distance for successive values on the axis 0x (Figure 3)
**^**	local minimum
**v**	local maximum
**_**	constant distance value for increasing values of 0x
	**Numerical parameters**
	angular value,
	maximum, minimum or fixed distance for specific 0x
	range of values on the axis 0x in which a given situation occurs

The alphabet has been adopted in clinical practice. However, its practical application is somewhat troublesome due to more complicated recording in comparison to conventional, previously known, methods (*TIA, TISA, AOD*). As a result, medical errors are more likely due to improper recording. Therefore, the authors suggested a new method, modified in relation to *AOS*, for measuring the iridocorneal angle, namely *AOM* (Angel Opening Minimum) which is described below.

## Material

In the study, about 100’000 tomographic images (from about 6’000 patients) were examined. The images were acquired using the following devices: SOCT Copernicus (Optopol Tech. SA, Zawiercie, Poland) and Visante OCT (Carl Zeiss Meditec, Inc, Dublin, California, USA). The patients, aged 12 to 78 years with varying degrees of the iridocorneal angle pathology, were from the region of Silesia, Poland. The obtained images were in DICOM or RAW formats with a resolution of 256 × 1024 pixels, within a measuring range of 8 mm × 16 mm, which gives 31.3 μm/pixel. The image analysis was carried out in a Matlab software package with Image and Signal Processing toolboxes and the code was optimized in the C programming language.

### *AOM* method

**Figure 5:**
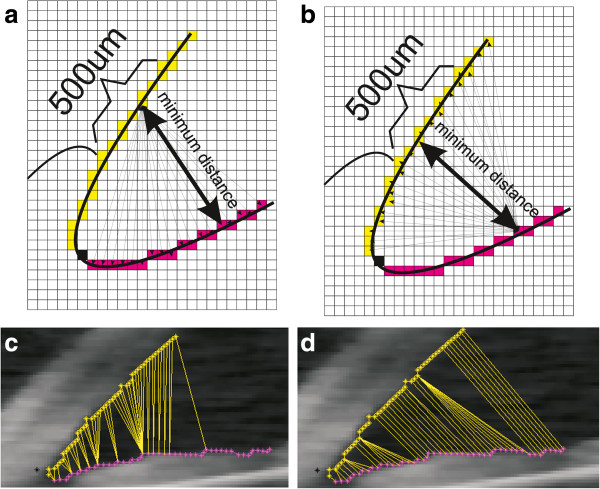
**The conception of *AOM *(Angle Opening Minimum) and *AOM2 *methods and the obtained results. a**) shows how the shortest distance between all the points of the cornea and one of the sample points of the iris is chosen in the *AOM* method. The shortest distance for both variants for one analysed pixel is highlighted in bold arrow. **b**) shows how the shortest distance between all the points of the iris and one of the sample points of the cornea is chosen in the *AOM2* method. **c**) and **d**) show practically obtained results for all pixels in the implementation of the two variants: the *AOM* method shown in **a**) and the *AOM2* method shown in **b**). In order to better visualize the results, the analysis was not limited to the appropriate range of 500 μm.

The *AOM* (Angle Opening Minimum) method involves determining a minimum distance, which is one scalar value, between the contours of the iris and cornea. To be more exact, it is the distance between different points of the cornea and the nearest point of the iris. The measured area of the cornea covers a range of 500 μm or 750 μm starting from the scleral spur. Details on the principle of the *AOM* method of measurement are shown in Figure 5a. Figure 5b, on the other hand, shows an opposite situation, where a minimum distance between various points of the iris and the nearest point of the cornea is calculated – the *AOM2* method. The *AOM* method is based on a sequence of calculations of the minimum angle values for individual pixels of the cornea edge from all the pixels of the iris edge (or the other way round in *AOM2* – Figure 5). Measurements can be carried out in the entire range for each of the contour points of the iris or cornea (as in Figure 5c and 5d) but only the contour points which are realized in the range of 500 μm of the corneal contour are relevant. This narrowing of the measurement range usually takes place in the last stage of calculations.

**Figure 6:**
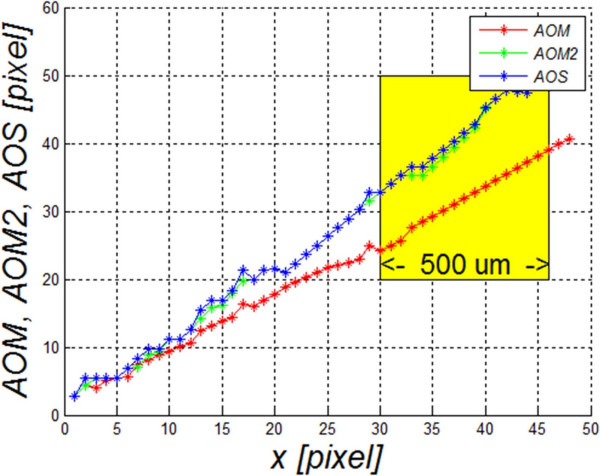
**Graph of changes in values of the angle opening for successive pixels of the contour of the cornea or iris, respectively (depending on the measurement method) shown in Figure**5**c) and d).** The graph shows the biggest difference between the *AOM* method and the other methods (*AOM2* and *AOS*). Visible differences are due to different measurement methods described in the paper. The area highlighted in yellow includes the range of 500 μm measured from the scleral spur (16 pixels · 31.3 μm/pixel ≅ 500 μm).

The obtained results differ depending on the measurement method (*AOS, AOM* or *AOM2* – Table 3). Examples of a sequence of distance measurements are shown in Figure 6. The graph shows changes in values of the angle opening for successive pixels of the contour of the cornea or iris, respectively, for the images in Figure 5c and 5d. The largest visible difference is between the *AOM* method and the other methods, namely *AOM2* and *AOS*. The difference is due to different measurement methods and amounts to approximately 10 pixels (10 pixels * 31.3 μm/pixel = 313 μm) in the range of 500 μm which is marked in yellow (Figure 6). In practice, due to a greatly variable shape of the contour of the iris in comparison with the contour of the cornea, the *AOM* method produces underestimated measurement values. However, they are more reliable when compared to the *AOS* or *AOM2* methods. They enable to take into account the iridocorneal angle pathology to a greater extent. Such situations concern the narrowing of the iridocorneal angle in the area outside the range of 500 μm when accurate (consistent with the definition) calculations with the methods *TISA, TIA* and *AOD* do not produce satisfactory results.

**Table 3:** Methods for measuring the iridocorneal angle proposed by the authors and their definitions

**Method symbol**	**Method name**	**Definition**
*AOS*	Angle Opening Sequence	(Figure 3) each iris contour point is combined with a suitable corneal contour point
*AOM*	Angle Opening Minimum	(Figure 5a) shortest distance between all the points of the cornea and selected range of the iris
*AOM2*	Angle Opening Minimum 2	(Figure 5b) shortest distance between all the points of the iris and selected range of the cornea

The result of the measurement of the iridocorneal angle with the *AOM* method is therefore a minimum distance between the iris and the cornea measured in the area of 500 μm starting from the scleral spur. The above mentioned range of 500 μm of the corneal contour (16 pixels fall into this range for the analysed image resolution) and all the pixels in the contour of the iris are taken into account in the measurements.

### Implementation of the *AOM* method

**Figure 7:**
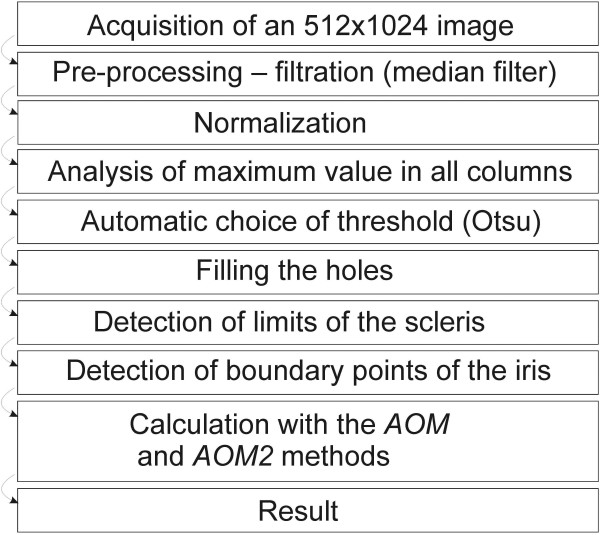
**Block diagram of the tomographic image analysis algorithm in the *AOM *method.** The presented algorithm enables fully automatic measurement of the iridocorneal angle. The obtained result is in the form of one scalar value which is the minimum distance between the selected ranges of the iris and cornea. The presented algorithm is versatile and provides correct results for any tomographic images of the eye.

The *AOM* method requires full automation of image analysis. This automation enables to detect the cornea and iris edges and then perform adequate calculations of the minima (according to the methodology of *AOM* described above). The image analysis algorithm, in particular, should include the following elements [9-12] shown in Figure 7. The need to use a profiled algorithm in this case is connected with inadequate results obtained with other known algorithms for detecting lines and/or areas in the image.

•Hough’s transform [7] enables to detect lines in images of a pre-selected shape. However, the results in the case of large inter-individual variability are not satisfactory.

•wavelet analysis method [13] gives incorrect results when objects are hard to see and the lines overlap – such situations are quite common in the case of the analysed images,

•analysis methods of elongated objects cannot be applied here due to a possibility of large changes in the size of both the object itself and its thickness and a possibility of its division into multiple parts (e.g. the iris or cornea).

•object recognition methods in cases of large pathology can give unpredictable results.

•other known methods, for example, texture analysis [14], also do not produce satisfactory results.

Based on this and the literature review [8,15-18] and given the medical evidence presented below, a profiled algorithm for the analysis and processing of images of the front part of the eye was suggested.

**Figure 8:**
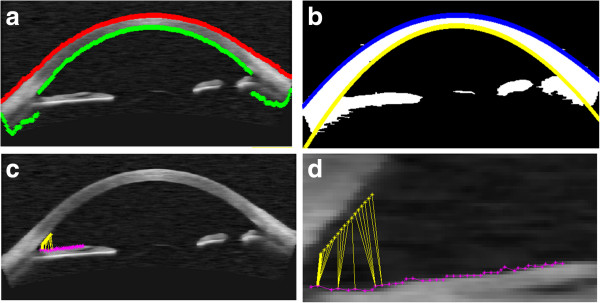
**Some stages of processing in the calculation of the iridocorneal angle with the *AOM *method: a) the sclera and cornea boundaries (in green and red) set automatically, b) approximation of the automatically set sclera boundary – in blue and yellow, c) the iridocorneal angle set automatically with the *AOM *method and its enlargement d).** According to the definition, the iridocorneal angle calculated with the proposed *AOM* method is a minimum distance calculated between the cornea pixels (the area of 500 μm) and all the pixels of the iris boundary. It is marked with a bold yellow line – **d**).

In the implemented algorithm, an input image with the resolution of 256 × 1024 pixels, mentioned in the introduction, is entered into the developed software in DICOM format. Then filtration with a median filter (with a 3 × 3 pixel mask) is carried out followed by the analysis of each column. As a result of this analysis, a binarization threshold is calculated for each column (Otsu method [19]). The created binary image is shown in Figure 8b). In the next stage, the method of filling the holes is applied in order to eliminate minor inclusions or detachment. In this pre-prepared image, the sclera boundaries are determined followed by approximation of the boundaries with a polynomial of degree 4 (Figure 8b). After the analyses of the iris, ciliary appendages and scleral spur, there follows the analysis of iris termination points which uses information from the inside of the sclera boundary (Figure 8c). At this stage, the iridocorneal angle can be determined using the *TIA, TISA* and *AOD* methods. The value of the iridocorneal angle measured with the *AOM* method (or *AOM2*) requires a calculation of Euclidean distance for each possible pair of points (iris-cornea). The result is a minimum distance between them calculated in the range of 500 μm, highlighted in Figure 8d in bold yellow.

This algorithm correctly identifies and calculates the iridocorneal angle not only with the *AOM* method but also with the other methods, namely *AOS, AOM2* or *TIA, TISA* and *AOD* at 500 and 750 μm from the scleral spur. All the algorithm parameters are calculated automatically. The algorithm automatically adjusts to the type and brightness of a derived image (pre-processing, filtering with a median filter with a 5 × 5 pixel mask or normalization). During the programme installation, an operator only gives a distance attributable to the pixel (strictly dependent on the type of the used tomographic camera) if it is not specified in the DICOM header. This information is necessary to calibrate the measured values of the iridocorneal angle (in the analysed images obtained with Visante OCT it is 31.3 μm/pixel).

Accuracy of the measurements and the comparison of the results obtained with the above methods (*AOD, TIA, TISA, AOS, AOM* and *AOM2*) are presented below.

### Comparison of *AOD*, *TIA*, *TISA*, *AOS*, *AOM* and *AOM2* methods in practice

A practical application of the measurement methods, namely *AOD, TIA, TISA, AOS, AOM* and *AOM2,* requires designation of the contours or a single point of the cornea and iris. In addition, it is necessary to determine the scleral spur location. The currently available software for OCT devices does not enable the mentioned fully automatic measurement. Calculations are carried out either manually or semi-automatically. For this reason, the results are not repeatable – they depend on an individual choice of the scleral spur (iridocorneal angle) location by an operator. The presented algorithm enables fully automatic measurement with the above mentioned methods. Therefore, their comparison is possible.

Of all the mentioned methods (*AOD, TIA, TISA, AOS, AOM* and *AOM2),* only AOD*, TIA, AOM2* and *AOM* are further taken into account. The *TISA* method is rarely used in practice due to the unit of the iridocorneal angle, that is, μm^2^ (mm^2^). The *AOS* method is also difficult to compare with other results because of a difficulty in comparison of an *AOS* data sequence (or the mentioned alphabet) with scalar values obtained as results from other methods.

The other methods, *AOD, TIA, AOM2* and *AOM*, can be compared using one of two ways. One of them uses a model (an artificial image) with a known and measured iridocorneal angle and the other one compares results with those obtained by ophthalmology experts. The first way, which will not be used here, is to use an artificial image containing known values of the iridocorneal angle realized with resolution error precision at the time of creating the image. The other way is to use the results obtained from an assessment performed by an ophthalmology expert or from other more accurate measurement methods (if they exist). Both ways have their advantages and disadvantages. The disadvantage of the first method is a difficulty in close conjunction of artificial images with real images which reflect a full range of variability. Its advantage is an ability to quickly compare the results and no need for the presence of an ophthalmologist. In the other method, results are obtained from a real image, but it requires a tedious procedure that involves manual marking (by an operator) of individual values of *AOD, TIA, AOM2* and *AOM*. This method is also highly dependent on the observer’s subjective judgment which is mainly related to the determination of significance of details in an image.

As a result, the comparison of the methods *AOD, TIA, AOM2* and *AOM* was carried out in two stages.

### Comparison with the results obtained by an expert

In the first stage, an ophthalmologist manually marked the iridocorneal angle for the first 100 images with the *AOD, TIA, AOM2* and *AOM* methods. In this case, a measurement error *δ*_*q*_ was calculated. It was defined as:

(1) $$\delta_{q}=\frac{w_{M}-w_{P}}{w_{P}}.100\%$$

where:

*w*_*M*_ – the measured value,

*w*_*P*_ – a correct value measured with more accurate methods (e.g. manually) or an average value of a series of measurements,

*q* – an index indicating the measurement method: *AOD, TIA, AOM2* or *AOM*.

The obtained values of *δ*_*q*_ are not greater than 5% for the *AOD* and *TIA* methods (δ_*AOD*_ = 4.5%, δ_*TIA*_ = 4.8%) and for *AOM* and *AOM2*, they are *δ*_*AOM*_ = 7.2% and *δ*_*AOM2*_ = 9%, respectively. Therefore, it seems that the presented *AOM* and *AOM2* methods are worse than the known methods. The reason for larger error values for these methods is the principle of measurement. It is much harder to manually select iris contour points located closest to the analysed corneal contour pixel (Figure 5) and then determine, of all the points, which of them is the smallest. Thus, it is necessary to compare the methods in terms of reliability of the iridocorneal angle calculation and detection of pathological cases. This methodology is described below.

### Comparison with the results obtained with other known methods

In the second stage, the comparison of the *AOD, TIA*, *AOM* and *AOM2* methods for all the obtained images (approximately 100’000) was performed by standardization of the measurement unit to relative values. This comparison was related to the effectiveness of detection of cases with normal and narrow iridocorneal angle, including pathological conditions. In order to compare the results, the following typical measures were used: accuracy *ACC*=(*TP*+*TN*)/(*TP*+*TN*+*FP*+*FN*), specificity *SPC*=*TN*/(*FP*+*TN*) and sensitivity *TPR*=*TP*/(*TP*+*FN*) where: *TP* – true positive, *TN* – true negative, *FN* – false negative and *FP* – false positive. These measures were used to evaluate the *AOD, TIA, AOM2* and *AOM* methods.

All cases where the iris or cornea was not visible were pre-eliminated. Such situations occurred when OCT imaging was not focused on calculating the iridocorneal angle. After removing such cases, 83'574 images remained for further analysis. It was carried out in the steps presented below.

#### Removal of all atypical cases for which the algorithm designated too short or too long iris edge

**Figure 9:**
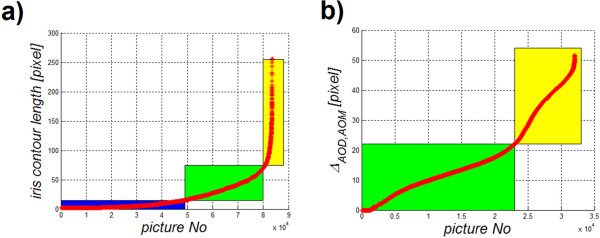
**Graph of changes in the iris length a) and the graph of changes in the value of *Δ***_***AOD,AOM***_**b) for the sorted (from the smallest value) images.** The area marked in blue in the graph **a**) covers the properly set iris contours but due to their length (<16 pixels, 500 μm) they cannot be used for further analysis. The acceptable values of the iris length are highlighted in green and the incorrectly designated iris contours in yellow. On the graph **b**), properly set values of the difference *Δ*_*AOD,AOM*_ are marked in green and the algorithm errors are in yellow.

Atypical cases are those for which the iris edge designation contains less than 16 pixels (500 μm, Figure 9a – in blue) or more than 70 to 80 pixels, which is equivalent to 2.5 mm (Figure 9a – in yellow). These limitations are natural and result from the presented methodology of measurement of *AOD, TIA, AOM2* and *AOM*. The distance from the scleral spur boundary used in the measurement is 500 μm, which corresponds to a minimum of 16 pixels. This limitation (16 pixels) arises only from the need to compare these methods, and smaller values (<16) do not indicate errors in the algorithm operation. There are 48'022 images of this type whose length of the iris contour is below 16 pixels. This represents 57.5% of the analysed images. Values above approximately 70 to 80 pixels of the iris edge length do not occur in medicine; therefore, they indicate an error in the algorithm operation. As is apparent from the graph showing the iris contour length for individual images, a number of such cases (exceeding 70 pixels) is relatively low (3'458, which is 4.1% of all cases). In total, 32’094 images remained for further analysis after applying these two limitations.

#### Comparison of the results obtained for the proposed AOM method and the AOD method

For this purpose, *Δ*_*AOD,AOM*_ was calculated as a difference between *AOD* and *AOM*. The results are shown in Figure 9b. The graph (Figure 9b) was divided in two parts. The changes in the value of *Δ*_*AOD,AOM*_ highlighted in green when compared to the *AOM* and *AOD* methods indicate undervaluation of the iridocorneal angle measurement for the *AOM* method. This situation is consistent with the definition according to which in the case of the *AOM* method, it is the smallest distance between the corresponding points in the iris and cornea that is sought. The part of the graph marked in yellow (Figure 9b) covers a range of pathological cases for which a direct comparison of *AOM* and *AOD* was not possible (incorrect location of the area of analysis for the *AOD* method or incorrect indication of a minimum in the *AOM* method). The graph of *Δ*_*AOD,AOM*_ as a function of a sequence of images was divided in two parts (Figure 9b):

•with differences between *AOD* and *AOM* arising from their definition (in practice to about 22 pixels) and,

•with differences between *AOD* and *AOM* caused by the algorithm errors.

The division between the two areas (the threshold value of 22 pixels) was verified in practice and included a manual analysis of borderline cases. This value also means that in carrying out a calculation for the afore mentioned group of images, the difference in the results for *AOD* and *AOM* methods will not be greater than 22 pixels for the most obtuse iridocorneal angle.

**Figure 10:**
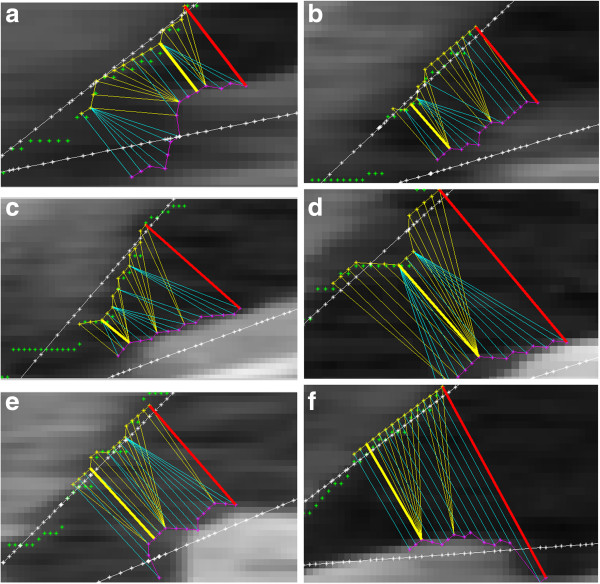
**Sample image fragments of the iridocorneal angle for which the known *AOD, TIA *and *TISA *methods give incorrect results.** For these examples, the iridocorneal angle is determined properly with *AOM* and *AOD* methods. The results obtained with the *AOM* method are marked with a bold yellow line, and the results obtained for the *AOD* method are in red. The other coloured lines refer to the cornea and iris contours (yellow, magenta), and sides of the angle of the *TIA* method (sides are shown as a linear interpolation of the iris and cornea contours) are marked in white. The results obtained for the examples are: **a**) *AOD* = 12.9, *AOM* = 7.8, *TIA* = 29.7 **b**) *AOD* = 15.6, *AOM* = 9.2, *TIA* = 27.1 **c**) *AOD* = 22.6, *AOM* = 6.5, *TIA* = 29 **d**) *AOD* = 26, *AOM* = 16, *TIA* = 24.5 **e**) *AOD* = 21.6, *AOM* = 15.9, *TIA* = 22.6 **f**) *AOD* = 30.2, *AOM* = 15.2, *TIA* = 29.8 (*AOM* and *AOD* values are given in pixels, *TIA* values in degrees).

As it turns out, in practice, the *AOM* method enables a proper evaluation of specific cases of the iridocorneal angle. These examples are shown in Figure 10. Thus it is possible to obtain correct classification of images with a proper, open iridocorneal angle from pathological cases with the risk of iridocorneal angle closure.

#### Comparison of quality of classification for the methods AOM, TIA and AOD

**Figure 11:**
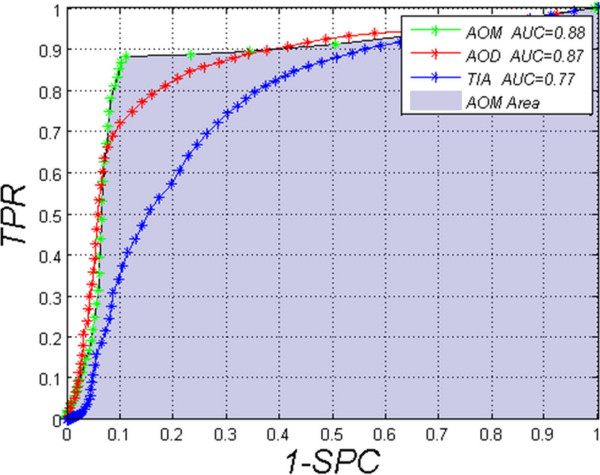
**ROC (Receiver Operating Characteristic) graph of the evaluation of classification for *TIA, AOD *and *AOM*.** The area under the curve for the best classifier of *AOM*, i.e. *AUC* = 0.88 is marked in blue. The other methods produce worse results i.e.: for *AOD*, *AUC* = 0.87 and for *TIA, AUC* = 0.77. Differences in the obtained classification quality result from the conception of measurement with *TIA* and *AOD* methods.

This comparison was made for a classification of healthy subjects and patients with the risk of iridocorneal angle closure (*TIA* < 15°, *AOD* < 190μm, *TISA* < 0.11 mm^2^[5,20]). Model values of the classification (due to the size of the group) were determined as the average of measurements performed with the *AOS* and *TISA* methods; additionally, in some cases, as the average of the results corrected by an expert. The division gave 20'536 cases with a correct iridocorneal angle and 2'454 cases with an incorrect narrowed angle. The *AOM, TIA* and *AOD* methods were compared for which the ROC (Receiver Operating Characteristic) curves shown in Figure 11 were obtained. For each method, the cut-off threshold was changed in the range from 0 to 180 by every 1, covering both the range of angular values (in the case of *TIA*) and the range of distance changes given in pixels (*AOM* and *AOD*). For the *TIA* method, optimal values were obtained for the threshold *TIA* = 18°, i.e.: *TPR* = 0.82 and *SPC* = 0.61. These are the worst results obtained for the compared methods. In this case, *AUC* is 0.77. The other compared method is *AOD*. In the case of this method, an optimal cut-off threshold is *AOD* = 8 pixels (about 240 μm). With this threshold value, *TPR* = 0.86 and *SPC* = 0.71 and *AUC* is 0.87. The last compared method is the new *AOM* method. It gave the best results for a threshold cut-off *AOM* = 6 pixels (about 180 μm) where *TPR* = 0.88, *SPC* = 0.89 and *AUC* = 0.88.

The adopted measurement definitions result from differences between the results. Distance point measurements are the main reason for errors in *AOD*. This method is very sensitive to noise and artefacts in images which can be observed especially in cases of high degrees of pathology. For the *TIA* method the situation is similar. A fixed point position of the angle measurement is very sensitive to noise. Another difficulty lies in finding an appropriate point of the angle apex [5,21,22]. Perhaps arranging the angle sides as a line approximating the iris and cornea contours would produce better results. The *TISA* method seems to be the most appropriate here. However, local narrowing of the iridocorneal angle enables to obtain the same results as in the case of slight angle narrowing, but in a larger area (500 μm) [23-26].

## Conclusions

The paper presents the comparison of known methods and proposes a new method of measuring the iridocorneal angle on the basis of tomographic images. This comparison shows that the best method is the one presented by the authors, namely *AOM* for which *AUC* = 0.88. This result is by 0.01 better than that obtained for the well-known *AOD* method (Tables 1, 2, 3). In terms of specificity, the difference between *AOD* and *AOM* is even greater (Figure 11) and amounts to 0.18. The differences between *AOD* and *AOM* result from specificity of measurement. For example, measurements for the *AOD* method (as well as for another method, i.e. *TIA*) are carried out pointwise [27-29]. The whole area of the iridocorneal angle, i.e. in the range of 500 μm starting from the scleral spur [5], is not taken into account. Moreover, minimum distance values are not calculated unlike in the *AOM* method. *AOM* enables to obtain reliable results in the form of a single scalar value in comparison with *AOS* (for which a sequence of values is obtained). Although *AOS* enables visualization of more complex cases (of the iridocorneal angle calculated for large pathology) in the form of a graph, it requires recording a series of numbers (which refer to distances between the retina and the cornea). One of the disadvantages of the *AOM* method is the need to perform calculations (of minimum distance) on a computer. Manual calculations of the iridocorneal angle with the *AOM* method may lead to worse results than in the case of the *TIA* and *AOD* methods [7,30,31].

In summary, this paper shows the advantage of automatic calculations of the minimum distance between the iris and the cornea within a specified range over the other previously known methods. The disadvantages of these known methods are: point measurement, lack of full automation or obtaining a series of information instead of a single number. The new proposed method is free of these defects. It enables to successfully diagnose pathological cases of iridocorneal angle narrowing, which is not possible with other available methods. It also enables to obtain very high repeatability of measurements and record the results in the form of single numbers. Owing to this method, implemented to any OCT device, ophthalmologists will receive more accurate results fully automatically – without any manual intervention. Not only will it increase comfort but it will also give rapid and reproducible results. In addition, it will enable to perform an automatic statistical analysis of a selected population of patients and monitor the progress of treatment, which will directly influence the costs of treatment, prevention and diagnosis of patients.

## Abbreviations

AOD: Angle opening distance; TIA: Trabecular-iris angle; TISA: Trabecular-iris space area; AOS: Angle Opening Sequence; AOM: Angle Opening Minimum; AOM2: Angle Opening Minimum 2; ROC: Receiver Operating Characteristic; ACC: Accuracy; SPC: Specificity; TP: True positive; TN: True negative; FN: False negative; FP: False positive

## Competing interests

The authors declare that they have no competing interests.

## Author contributions

RK and ZW suggested the algorithm for image analysis and processing, implemented it and analysed the images. SW, AN, EW performed the acquisition of the OCT images and consulted the obtained results. All authors have read and approved the final manuscript.
